# Dosimetric advantage of volumetric modulated arc therapy in the treatment of intraocular cancer

**DOI:** 10.1186/s13014-017-0819-7

**Published:** 2017-05-10

**Authors:** Zhenxiang Deng, Lanxiao Shen, Xiaomin Zheng, Yongqiang Zhou, Jinling Yi, Ce Han, Congying Xie, Xiance Jin

**Affiliations:** Radiotherapy and Chemotherapy Department, the 1st Affiliated Hospital of Wenhzou Medical University, No.2 Fuxue Lane, Wenzhou, 325000 China

**Keywords:** Intraocular cancer, Conformal radiotherapy, Intensity-modulated radiotherapy, Volumetric-modulated radiotherapy

## Abstract

**Objective:**

The purpose of this study is to investigate the dosimetric advantages of volumetric modulated arc therapy (VMAT) in the treatment of intraocular cancer by comparing it directly with three-dimensional conformal radiotherapy (CRT) and intensity-modulated radiotherapy (IMRT).

**Methods:**

CRT plan, 7f-IMRT plan, and one-arc VMAT plan were generated for 14 intraocular cancer patients. Dosimetric and biological quality indices for target volume and organs at risks (OARs) were evaluated and compared.

**Results:**

The target coverage presented by V95 for CRT, IMRT and VMAT were 95.02% ± 0.67%, 95.51% ± 2.25%, and 95.92% ± 3.05%, respectively. The homogeneity index (HI) for CRT, IMRT and VMAT were 0.15 ± 0.05, 0.23 ± 0.05, and 0.23 ± 0.06, respectively. IMRT and VMAT greatly decreased the dose to ipsilateral lens compared with CRT with a D1 of 2972.66 ± 1407.12 cGy, 3317.82 ± 915.28 cGy and 4809.54 ± 524.60 cGy for IMRT, VMAT and CRT, respectively. Similar results were observed for ipsilateral eyeballs. IMRT and VMAT also spared better on brainstem, optical nerves and optical chiasm compared CRT. However, CRT achieved lower dose to the eyeballs compared with IMRT and VMAT. VMAT and IMRT showed mixed results on target coverage and OAR sparing. The average MUs and delivery time of IMRT and VMAT were 531.25 ± 81.21 vs. 400.99 ± 61.49 and 5.05 ± 0.53 vs.1.71 ± 0.69 min, respectively.

**Conclusions:**

Although no clear distinction on PTV coverage among CRT, IMRT and VMAT plans was observed in the treatment of intraocular cancer, VMAT and IMRT achieved better homogeneity and conformity for target volume, and delivered fewer doses to ipsilateral lens and eyeballs compared with CRT. However, VMAT and IMRT increased the low dose volume to the contralateral OARs. Although VMAT and IMRT showed mixed results on target coverage and OAR sparing, VMAT decreased MU and delivery time significantly compared with IMRT. VMAT is a promising and feasible external beam radiotherapy technique in the treatment of intraocular cancer patients.

## Introduction

Intraocular cancer, which includes primary and secondary intraocular cancers, presents a therapeutic challenge due to the sensitive tissues involved and the necessity to destroy the tumor while minimizing visual loss. Primary intraocular cancers start inside the eyeball. In adults, melanoma is the most common primary intraocular cancer. In children, retinoblastoma (a cancer arising from cells in the retina) is the most common primary intraocular cancer, and medulloepithelioma is the next most common [1]. Secondary intraocular cancers start somewhere else and then spread to the eye, which are actually more common than primary intraocular cancers. The most common cancers that spread to the eye are breast and lung cancers [2].

Local therapy options for management of intraocular disease include enucleation, radiation therapy (RT), cryotherapy, and laser therapy [3]. Radiation therapy (RT) had been well described in the management of orbital lymphoma [4–8]. External beam radiation therapy (EBRT) is currently considered the most common treatment modality for intraocular cancer, which provides lower late recurrence rates with respect to radioactive plaque brachytherapy [9]. EBRT also has an advantage over surgery by preserving the eye structure, which may result in a better appearance after treatment. The main concern with radiation therapy is damage to parts of the eye, leading to problems such as cataracts, retinal detachment, glaucoma, or bleeding into the eye [10–12].

The delivery of radiotherapy to orbit is technically challenging given the critical structures in the treatment field and their relatively low tolerance levels. In the past, a single enface electron beam or AP beam was used in the radiotherapy. The radiation is often delivered using wedged anterior and lateral fields directed at the target volume. This technique causes significant fluctuations in dose homogeneity within the treatment field, often with hotspots of more than 25%. The conventional RT often causes acute side effects in many patients and induces dry eye syndrome and conjunctivitis [4]. A dose reduction to the critical structures during radiotherapy had been a concern of physicians.

Advances in RT technology, such as proton therapy, intensity-modulated radiation therapy (IMRT) and volumetric modulated arc therapy (VMAT) allow more conformal dose distributions for patients with intraocular cancer [13, 14]. The unique dosimetric properties of IMRT and VMAT have the potential to reduce the injury to uninvolved structures while attaining appropriate tumor coverage and may lead to an improved therapeutic index with respect to tumor control and toxicity [15–17]. Particularly, VMAT technique had gained enormous interest world-wide by using continuous changing MLC movement, gantry rotation and dose rate with less MUs and delivery time [18, 19]. VMAT improves dose homogeneity and sparing of critical organs over IMRT for many tumor sites [16, 17, 19].

Eldebawy et al compared the dosimetric distributions among radiotherapy techniques, including electron beam, photon beam with wedge pair, 3D-CRT, IMRT, VMAT, fractionated stereotactic radiotherapy, and helical tomotherapy in three retinoblastoma patients. They concluded that inverse planned image-guided radiotherapy using tomotherapy or VMAT obtained a better conformity index, a lower integral dose and improved orbital bone and brain sparing compared with other techniques [20]. Except for this study, few further study had been carried out to explore the dosimetric advantage of VMAT in the treatment of intraocular cancer patients. The purpose of this study is to investigate the dosimetric advantages of VMAT in the treatment of intraocular cancer by comparing it with CRT and IMRT.

## Materials and methods

### Patients and simulation

Fourteen patients with confirmed primary and secondary intraocular cancer were enrolled in this study. Patients were immobilized in supine position using a thermoplastic mask system with active fixation of light points and scanned with a Philips Brilliant spiral CT (Philips Brilliant, Cleveland, OH) according to standard procedures with 3 mm slice spacing [21]. MR images in T1 and T2 phases were obtained at 3 mm slice spacing to facilitate the target delineation.

### Target contour and planning

Gross target volume (GTV) was defined as the gross extent of tumor demonstrated by CT and MRI imaging studies. Planning target volume (PTV) was delineated with a 3 mm margin from GTV. Normal tissue structures were also contoured by one experienced radiation oncologist on the CT dataset on a slice-by-slice basis, including the right and left lens, right and left eyeballs, optic nerves, optic chiasm and brainstem.

The goal of treatment planning was to get a good coverage of PTV while sparing normal tissues. For the sake of dosimetric comparison, prescription was normalized to 50 Gy at 25 fractions at 6MV for all the patients and plans. CRT, 7-filed IMRT and one-arc VMAT plans were generated for each patient using Elekta Monaco treatment planning system (Clinical version 5.1.1, Elckta, UK). For CRT plans, three coplanar beams were manually selected and calculated with collapsed cone convolution (CC convolution) algorithm. Monte Carlo algorithm was applied to optimize the final dose of IMRT and VMAT plans. All plans were optimized to reach clinically acceptable PTV coverage and organ at risk (OAR) sparing. At least 95% of the PTV must be covered by 95% of the prescription dose.

For the planning, the optimization constraints based on the biological cost functions (i.e. Serial or parallel complication model for OARs and Poisson cell kill function for the PTV). For final Monte Carlo dose calculations, a calculation grid of 3 mm and a 1%variance were used. All plans were normalized to the 95% isodose line encompassing 95% of the PTV (V95% = 4750 cGy)..

### Plan evaluation and comparison

Quantitative evaluation of plans was performed by means of standard dose–volume histogram (DVH). For PTV, the values of D99% and D1% (dose received by the 99% and 1% of the volume) were defined as metrics for minimum and maximum doses and consequently reported. V95% (the volume receiving at least 95% of the prescribed dose) was reported as the target coverage. Homogeneity index (HI) was evaluated as the difference between D1 and D99 of PTV and divided by the prescription dose (Dp) [22]:

1 $$ \mathrm{H}\mathrm{I}=\frac{\mathrm{D}1-\mathrm{D}99}{\mathrm{D}\mathrm{p}}\times 100\% $$

Conformity index (CI) [23] was also calculated for PTV:

2 $$ C I=\frac{V_{T, Pi}}{V_{{}_{Pi}}} $$

Where V_T,Pi_ is the volume of PTV covered by the prescription isodose, and V_Pi_ is the volume of the body that is covered by the prescription isodose. The maximum value of CI is 1, corresponding to a perfect coverage of PTV.

Radiobiological indices of tumor control probability (TCP) and normal tissue complication probability (NTCP) were also calculated using the Niemierko model [24]. The equivalent uniform dose (EUD) was obtained as an expectation value:

3 $$ E U D={\left(\frac{1}{N}{\displaystyle \sum_1^N}{D}_i^a\right)}^{\frac{1}{a}} $$

Where N is the number of voxels in the structure of interest, D_i_ is the dose in the ith voxel, and α is the tumor normal tissue-specific parameter that describes the dose-volume effect. Based on the EUD, the TCP can be calculated by

4 $$ \mathrm{T}\mathrm{C}\mathrm{P} = \frac{1}{1+{\left[\mathrm{TCD}50/\mathrm{EUD}\right]}^{4\upgamma 50}} $$

Where TCD50 is the tumor dose required to produce 50% TCP, *γ*50 is the slope of dose response at 50% TCP. The tumor-specific parameters were cited from the study of Okunieff et al [25].

For OARs and health tissues, the analysis included the mean dose and a set of appropriate V_X_ and D_Y_ values. In the case of biological analysis, the NTCP is determined as

5 $$ NTCP = \frac{1}{1+{\left[ TD50/ EUD\right]}^{4\gamma 50}} $$

Where TD50 is the dose at which the probability of complication becomes 50% in 5 years and *γ*50 is the slope of signoidal dose-response curve of normal tissue at complication probability. These tissue-specific parameters are based on the Niemierko model [24]. The TCP of PTV and NTCPs of brainstem, lens, eyeball, optic nerves and optic chiasm were calculated for plan evaluation. Parameters applied in this study for TCP and NTCP calculation were summarized in Table 1.

**Table 1:** Parameters for TCP and NTCP calculation cited

	Tumor	Lens	Brainstem	Eyeball	Optic nerves	Optic chiasm
TCD50 (Gy)	51.77					
γ50	2	1	3	2	25	25
α	−13	3	7	15	3	3
TD50/5		18	65	65	65	65

MU, delivery time and gamma passing rate for IMRT and VMAT were also evaluated and compared. VMAT and IMRT QA were performed using a 3D diode array ArcCHECK (Model 1220) and SNC Patient v. 6.2.1 (Sun Nuclear Corporation) with a global gamma passing criteria of 3%/3 mm and 10% lower dose threshold [26]. All plans were delivered through a MosaiQ® record and verify system v. 1.60Q3 (IMPAC Medical Systems, Inc., Sunnyvale, CA) on an Elekta Synergy® linac (Elekta Ltd, Crawley, UK) equipped with an 80-leaf MLCi2™.

### Statistical analysis

Results were described as mean ± standard deviation (SD). Comparison of dosimetric and nondosimetric indices among plans with different treatment modalities were analyzed with Wilcoxon signed rank test. All statistical analysis was conducted with R program software. Difference was considered statistically significant when *p <* 0.05.

## Results

Table 2 presents the characteristics of the enrolled 14 patients with primary and secondary intraocular cancers. There were 5 female and 9 male patients with a median age of 55 (range from 33-78 years old). Total of 52 plans were generated for these patients.

**Table 2:** Patient characteristics

Patient	Sex	Age	Diagnosis	Location	PTV volume (cm^3^)
1	Male	47	Malt lymphoma	Left eye	20.88
2	Male	43	Orbital adenocarcinoma	Left eye	48.50
3	Male	55	Malt lymphoma	Left eye	31.85
4	Male	47	Orbital tumor	Right eye	38.45
5	Male	49	Orbital tumor	Right eye	11.65
6	Female	80	Eyelid carcinoma	Left eye	28.89
7	Male	56	Malt lymphoma	Right eye	22.89
8	Female	59	Lung metastasis	Left eye	83.74
9	Male	65	Malt lymphoma	Right eye	46.11
10	Female	41	Malt lymphoma	Left eye	10.11
11	Male	59	Malt lymphoma	Right eye	14.97
12	Male	56	Malt lymphoma	Left eye	20.84
13	Female	33	Orbital adenocarcinoma	Left eye	8.87
14	Female	78	Malt lymphoma	Right eye	27.34

**Fig. 1:**
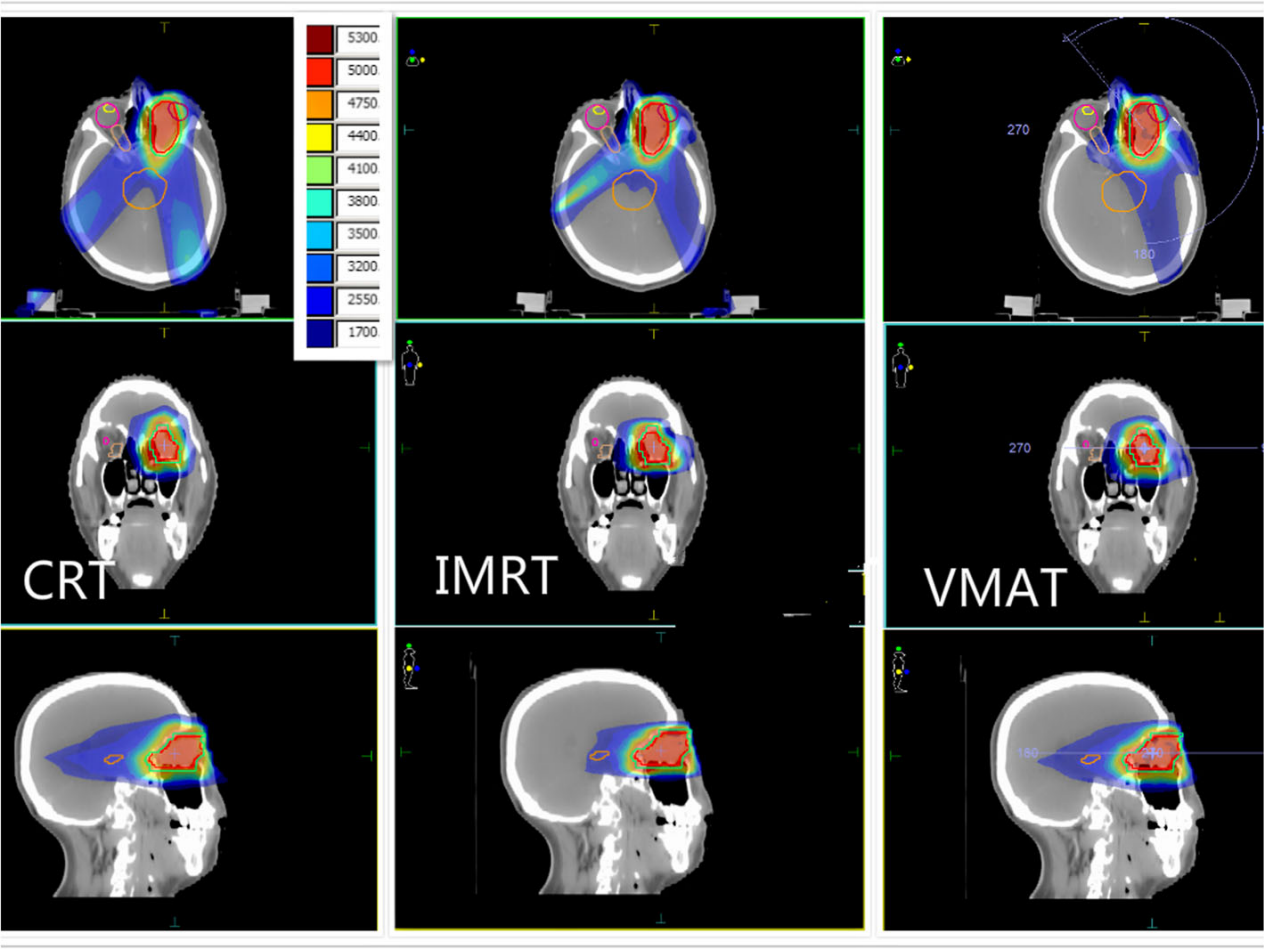
Typical dose distribution comparison among CRT, IMRT and VMAT for one intraocular cancer patient

**Fig. 2:**
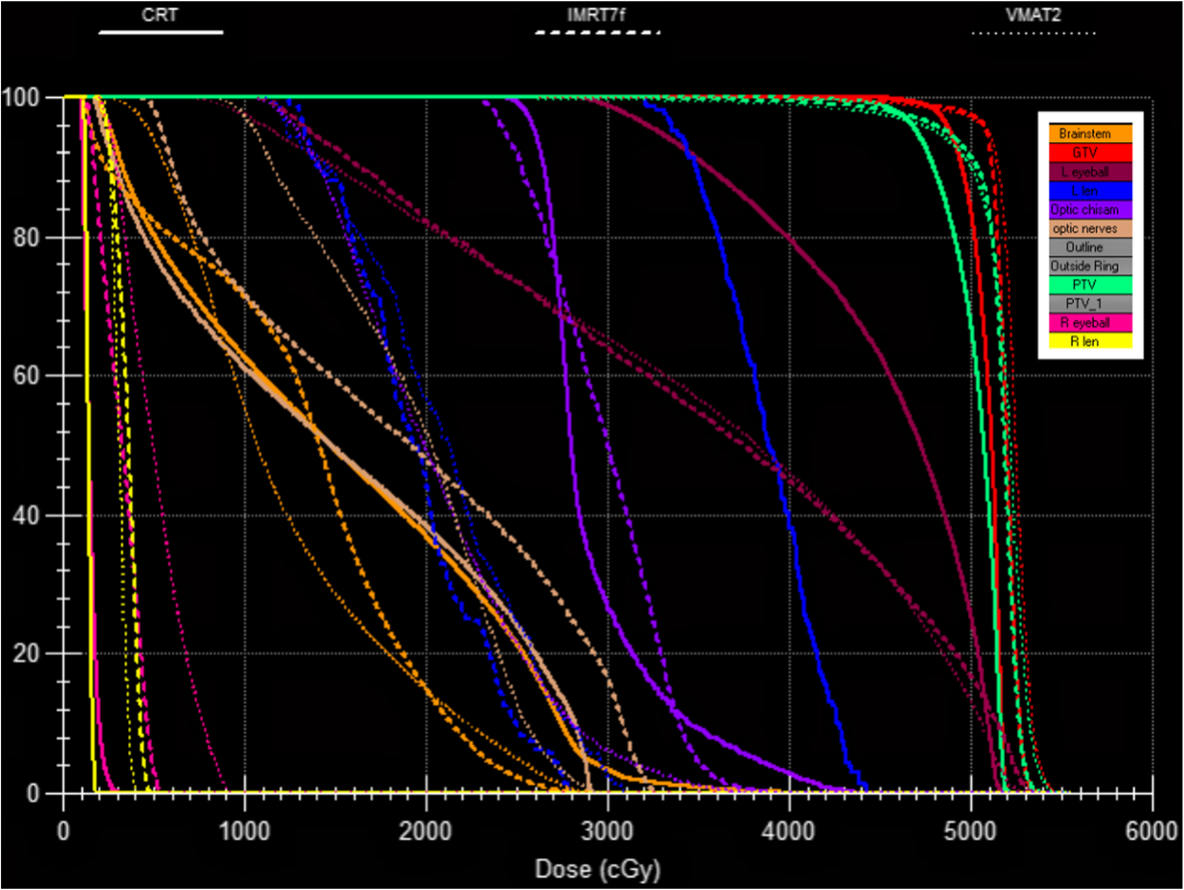
Typical DVH comparison among CRT, IMRT and VMAT for one intraocular cancer patient

A typical dose distribution comparison and DVH comparison were shown in Figs. 1 and 2. The high dose volumes of IMRT and VMAT matched better to the target volume compared with CRT. Detailed dosimetric comparison on target coverage was presented in Table 3. The target coverage of PTV for three modalities were all clinical acceptable with a V95 of 95.02% ± 0.67%, 95.51% ± 2.25%, 95.92% ± 3.05% for CRT, IMRT, and VMAT, respectively. The HI of the CRT, IMRT, and VMAT were 0.15 ± 0.05, 0.23 ± 0.05, and 0.23 ± 0.06, respectively.

**Table 3:** Target coverage comparison

PTV	CRT	IMRT	VMAT	*P-*value		
				CRT vs IMRT	CRT vs VMAT	IMRT vs VMAT
Dmean(cGy)	5065.87 ± 111.52	5175.76 ± 38.45	5157.21 ± 52.23	<0.01	0.04	0.20
V95 (%)	95.02 ± 0.67	95.51 ± 2.25	95.92 ± 3.05	0.48	0.91	0.30
D95(cGy)	4750.5 ± 14.25	4789 ± 150.20	4756.86 ± 132.03	0.37	0.86	0.32
D1(cGy)	5303.43 ± 175.25	5422.32 ± 35.59	5425.00 ± 33.59	0.02	0.03	0.84
D99(cGy)	4540.50 ± 109.96	4274.62 ± 277.51	4268.57 ± 304.07	<0.01	0.01	0.92
HI	0.15 ± 0.05	0.23 ± 0.05	0.23 ± 0.06	<0.01	<0.01	0.89
CI	0.94 ± 0.01	0.95 ± 0.02	0.95 ± 0.03	0.23	0.87	0.29
EUD(cGy)	4791.42 ± 834.88	4896.8 ± 408.80	4824.45 ± 423.00	0.47	0.881	0.52
TCP	0.90 ± 0.25	0.94 ± 0.10	0.95 ± 0.09	0.39	0.67	0.88

Table 4 lists the OARs protection comparison among three planning modalities. The D1 of the ipsilateral eyes were 4809.54 ± 524.60, 2972.66 ± 1407.12, and 3317.82 ± 915.28 (cGy) for CRT, IMRT and VMAT, respectively. Significant differences were observed between CRT vs. IMRT (*p <* 0.01) and CRT vs. VMAT (*p <* 0.01), but not with IMRT vs. VMAT (*p =* 0.50). The Dmean of the ipsilateral and contralateral eyeball were 4809.54 ± 524.60, 2972.66 ± 1407.12, 3317.82 ± 915.28, and 214.77 ± 279.60, 462.76 ± 900.98, 436.87 ± 186.89 (cGy) for CRT, IMRT and VMAT, respectively. Detailed comparisons for other OARs were shown in Table 4.

**Table 4:** OAR sparing comparison

OAR	CRT	IMRT	VMAT	*P-*value		
				CRT vs IMRT	CRT vs VMAT	IMRT vs VMAT
Ipsilateral lens						
D1(cGy)	4809.54 ± 524.60	2972.66 ± 1407.12	3317.82 ± 915.28	<0.01	<0.01	0.50
EUD(cGy)	4339.37 ± 811.97	2299.64 ± 787.15	2390.57 ± 733.59	<0.01	<0.01	0.30
NTCP	0.95 ± 0.08	0.65 ± 0.26	0.70 ± 0.26	<0.01	<0.01	0.13
Ipsilateral eyeball						
Dmean(cGy)	4558.41 ± 455.76	3071.33 ± 920.03	3087.5 ± 386.25	<0.01	<0.01	0.23
D1(cGy)	5253.86 ± 162.60	4976.07 ± 1321.53	5239.64 ± 130.72	0.46	0.82	0.46
EUD(cGy)	4922.18 ± 223.88	4680.53 ± 159.80	4579.88 ± 217.23	<0.01	<0.01	0.02
NTCP	0.10 ± 0.04	0.07 ± 0.02	0.06 ± 0.02	<0.01	<0.01	0.02
Contralateral lens						
D1(cGy)	272.18 ± 345.84	946.63 ± 1314.73	382.71 ± 110.54	0.10	0.23	0.13
EUD(cGy)	217.28 ± 296.92	211.97 ± 129.58	299.96 ± 89.88	0.95	0.29	0.02
Contralateral eyeball						
Dmean(cGy)	214.77 ± 279.60	462.76 ± 900.98	436.87 ± 186.89	0.35	<0.01	0.89
D1(cGy)	412.96 ± 508.90	887.93 ± 1324.14	978.18 ± 445.59	0.23	<0.01	0.80
EUD(cGy)	346.45 ± 422.16	448.63 ± 315.48	798.11 ± 361.61	0.31	<0.01	<0.01
Brainstem						
D1(cGy)	2615.36 ± 1026.22	1820.59 ± 709.32	1401.00 ± 606.04	0.02	<0.01	<0.01
EUD(cGy)	2029.99 ± 905.89	1321.18 ± 552.40	1001.44 ± 421.73	0.01	<0.01	<0.01
Optic nerves						
D1(cGy)	4886.14 ± 644.38	4444.54 ± 825.25	4532.32 ± 854.19	0.03	0.07	0.57
EUD	3356.65 ± 665.49	2592.39 ± 719.44	2973.78 ± 772.57	<0.01	0.03	0.03
Optic chiasm						
D1(cGy)	1513.89 ± 1082.09	1442.11 ± 1264.59	1456.07 ± 892.92	0.87	0.86	0.97
EUD(cGy)	2256.45 ± 907.36	1646.8 ± 995.51	1396.54 ± 646.45	0.04	0.04	0.25

The average MUs of CRT, IMRT and VMAT were 235.65 ± 44.32, 531.25 ± 81.21 and 400.99 ± 61.49 (*p <* 0.01), respectively. The delivery time for CRT, IMRT and VMAT were 2.71 ± 0.32, 5.05 ± 0.53 and 1.71 ± 0.69 min (*p <* 0.01), respectively. The percentage gamma pass ratios of IMRT and VMAT were 98.86% ± 1.03% and 98.93% ± 0.98% (*p =* 0.88), respectively.

## Discussion

The dosimetric advantages of VMAT in the treatment of primary and secondary intraocular cancer were investigated by comparing it directly with CRT and IMRT in this study. VMAT and IMRT increased the homogeneity and TCP for PTV compared with CRT, although no other target coverage difference was observed. VMAT and IMRT decreased the dose to ipsilateral lens compared with CRT. However, they also increased the low dose volume to the contralateral OARs.

Due to the vicinity of critical organs, such as the lens, optic nerve, optic chiasm, etc, optimizing the dose coverage on target volumes while sparing critical organs has been a challenge in the radiotherapy of intraocular cancer. Previous 2D and 3D RT techniques with a weighted anterior field and/or two wedged lateral fields were used to avoid irradiation on eyes and to overcome the dose heterogeneity. However, the dose distribution was usually negatively affected by the complex tumor shape and tissue heterogeneity [20]. In this study, although there was no significant difference on target coverage (V95) among CRT, IMRT and VMAT observed, IMRT and VMAT increased the mean dose and maximum dose to target compared with CRT. But IMRT (*p <* 0.01) and VMAT (*p <* 0.01) resulted a higher HI compared with CRT, indicating a worse dose homogeneity. There was no significant difference on target coverage observed between IMRT and VMAT. Similar results between IMRT and VMAT had been reported in head-and-neck cases and prostate patients [15, 27].

In this study, VMAT (*p <* 0.01) and IMRT (*p <* 0.01) plans significantly decreased the maximum dose (D1) and EUD to the ipsilateral lens, as well as the dose delivery to other ipsilateral OARs compared with CRT plans. Similarly, Goyal el at. demonstrated the superiority of IMRT plan in sparing the ipsilateral OARs compared with CRT plan for intraocular cancer [28]. As for the dose delivery to contralateral OARs, IMRT shown an increase on D1 to contralateral lens and eyeballs compared with CRT but without statistical significance. VMAT showed a significant dose increases to contralateral eyeball compared with CRT. This increased large volume of low dose irradiation on contralateral eyeball might be the cost of increased dosimetric homogeneity achieved by IMRT and VMAT.

In this study, VMAT showed a higher EUD on contralateral eyes, lens and optical nerves compared with IMRT. Although VMAT delivered less dose to ipsilateral eyeball, optical chiasm and brainstem than IMRT, only the EUD and NTCP of ipsilateral eyeball showed a significant difference (both *p =* 0.02). These mixed results between IMRT and VMAT were consistent with previous comparing studies indicating that IMRT and VMAT were equally superior in the target coverage and OAR sparing, the differences may basically depend on different cases and different priorities on optimization parameters selected by planners during optimization. In a study of Bertelsen et al in the treatment of head-and-neck cancer, VMAT improved the CI compared with IMRT. While in a study of Vanetti et al, it indicated that IMRT and VMAT plan were equivalent in terms of CI [15, 27].

In this study, VMAT plans decreased the mean MU and delivery time greatly compared with IMRT. This was consistently reported in previous studies [16, 17]. It had been reported that the increased MUs and leakage radiation in IMRT lead to an increase of radiation induced secondary malignancies [29]. The decreased delivery time achieved by VMAT could have a clinical impact on patients in terms of comfort on the couch and an increase of patient throughout. It will help to decrease the interfraction errors for patients with intrinsic movement [27].

## Conclusions

VMAT and IMRT achieved better homogeneity and conformity for target volume, and delivered less dose to ipsilateral lens and eyeballs compared with CRT in the treatment of intraocular cancer patients. However, VMAT and IMRT increased the low dose volume to the contralateral OARs. Although VMAT and IMRT showed mixed results on target coverage and OAR sparing, VMAT decreased MU and delivery time significantly compared with IMRT. VMAT is a promising and feasible external beam radiotherapy technique in the treatment of intraocular cancer patients.

## Abbreviations

CI: Conformity index
CRT: Conformal Radiation Therapy
DVH: Dose-volume histogram
EUD: Equivalent uniform dose
GTV: Gross target volume
HI: Homogeneity index
IMRT: Intensity-modulated radiation therapy
NTCP: Normal tissue complication probability
OAR: Organ at risk
PTV: Planning target volume
TCP: Tumor control probability
VMAT: Volumetric modulated arc therapy
